# The State of Knowledge about Nutrition Sources of Vitamin D, Its Role in the Human Body, and Necessity of Supplementation among Parents in Central Poland

**DOI:** 10.3390/ijerph15071489

**Published:** 2018-07-14

**Authors:** Katarzyna Zadka, Ewelina Pałkowska-Goździk, Danuta Rosołowska-Huszcz

**Affiliations:** Department of Dietetics, Faculty of Human Nutrition and Consumer Sciences, Warsaw University of Life Sciences WULS-SGGW, 159c Nowoursynowska ST., 02-776 Warsaw, Poland; ewelina_palkowska_gozdzik@sggw.pl (E.P.-G.); danuta_rosolowska_huszcz@sggw.pl (D.R.-H.)

**Keywords:** vitamin D, supplementation, nutrition sources, parents’ knowledge

## Abstract

The percentage of children with vitamin D deficiency in Poland is alarming. The aim of the study was to assess the knowledge about sources of food and the function of vitamin D, as well as the frequency of its supplementation. A survey was conducted among the parents of children from Central Poland attending primary schools, and a questionnaire containing mainly open-ended questions was used to collect the data. Most mothers knew at least one of the functions of vitamin D in the body but had a low level of knowledge about its dietary sources. Only a small group of respondents supplemented themselves and their children with vitamin D. Statistically significant influences on the level of knowledge about the functions and sources of vitamin D were place of residence (i.e., better knowledge in the countryside) and mothers’ level of education (i.e., the better educated, the greater knowledge). In the case of monthly income level, such impact was observed only in relation to the knowledge of vitamin D functions. Concerning the frequency of supplementation, only maternal level of education had a statistically significant effect (i.e., the higher the education level, the higher the frequency of supplementation). In addition, mothers who were aware of functions of vitamin D and nutritional sources, significantly more frequently supplemented vitamin D.

## 1. Introduction

The results of epidemiological studies published over the past few years indicate that vitamin D deficiencies affect almost a billion people in the world and are associated with the occurrence of many non-communicable diseases. Hypovitaminosis of vitamin D can be a result of a limited exposure to sunlight, using sunscreen, air pollution, insufficient intake of foods rich in this vitamin and/or problems with its absorption [1]. Vitamin D deficiency is a common medical condition in Poland that affects 50–80% of adults [2], and the prevalence of vitamin D insufficiency among children and adolescents reaches even 80% [3].

Vitamin D has multiple physiological roles. Its primary function is to maintain homeostasis of calcium and phosphorus, which is necessary for proper bone mineralization. Therefore, rickets among children, and osteomalacia and osteoporosis among adults, are the most often mentioned effects of its deficiencies [4]. Vitamin D increases the ability of small intestinal epithelial cells to absorb calcium and regulate the absorption of phosphorus from food, and also stimulates the re-adsorption of calcium from glomerular filtrate [5]. Vitamin D affects the muscular system by stimulating the proliferation and differentiation of muscle cells [6]. Its deficiency is also strongly correlated with the occurrence of neurodegenerative disorders such as: Schizophrenia, senile dementia or multiple sclerosis [7]. In addition, the stimulating effect of the renin-angiotensin-aldosterone system points to the mechanism connecting vitamin D deficiency with the development of cardiovascular diseases [8].

Vitamin D also has a significant effect on the immune system. Its adequate supply prevents respiratory infections and indirectly participates in the production of compounds with antibiotic properties (i.e., cathelicidin and defensin) [9]. The optimal level of vitamin D has a positive effect on the condition and healthiness of the skin, and the regulation of reproductive processes in both women and men [10]. In addition, association has been found between low UVB irradiance and high incidence rates of type 1 diabetes during childhood, which provided new support for the concept of the role of vitamin D in reducing the risk of type 1 diabetes [11]. Therefore, vitamin D supplementation among children could help to reverse the increasing trend in the incidence of this disease [12]. It should be noted that the higher serum level of 25-hydroxyvitamin D is associated with substantially lower incidence rates of colon, breast, ovarian, renal, pancreatic, aggressive prostate and other types of cancer [13]. Vitamin D concentrations above 40 ng/mL was found to be related to substantial reductions in risk of all cancers combined [14]. Inadequate vitamin D supply may also play a role in the pathogenesis of chronic infectious diseases, autoimmune diseases, allergies and psychiatric disorders [15].

Currently, the recommendation of vitamin D supplementation for healthy children and adolescents (aged 1–18) is 600–1000 IU per day (depending on body mass) from October to April, or during the whole year if dermal synthesis is not sufficient [16]. In case of high-risk groups of deficiency, 1000 IU per day is recognized as a safe level of supplementation for infants, and supplementation of up to 10,000 IU per day is considered safe for adults, including nursing mothers [17]. Despite existing national and worldwide guidelines concerning supplementation, deficiency of vitamin D is still common among all age groups. The natural consequence of this situation should be an implementation of actions aimed at increasing awareness of society and the medical community about the role of vitamin D, and how important it is to mitigate its deficit [18].

Although there are many studies regarding the effects of vitamin D deficiency in various age groups and the impact of vitamin D on health, there is limited research concerning knowledge and the popularity of supplementation in Poland, and none of this research examined knowledge and supplementation at the same time. A survey of 118 parents of children aged 4–6 carried out in 2014 showed insufficient vitamin D supplementation among children. During the whole year, only 14.4% of the observed pre-school children received it daily and cod liver oil was the most commonly chosen supplement. Despite the lack of verification of the level of knowledge about vitamin D among parents, authors concluded that on-going education of parents concerning nutrition principles of children is necessary [19]. A study assessing the state of knowledge about vitamin D performed among 400 adult hospital patients in Lodz (one of the most populated cities in Poland), demonstrated the inability of 93% of them to indicate the beneficial influence of vitamin D on their health, including protective effects on the digestive system during chemotherapy. Almost all respondents complained that they had not been informed about the role of this component on their treatment [20].

In our opinion, the wider recognition of the level of knowledge about vitamin D in Poland is critical to develop an effective method of education aimed at improving its intake, both in children and adults. Therefore, the aim of our study was to evaluate the knowledge concerning food sources and the function of vitamin D among parents of children attending primary schools in Central Poland, as well as the frequency of supplementation of this vitamin among both parents and children. The relationship between the level of knowledge and the frequency of supplementation in relation to selected environmental factors was also assessed, including: Place of residence, income, education, and mother’s weight. These socio-economic status indicators were chosen because of the notable influence parents’ lifestyle (such as food intake) has on children’s and adolescents’ dietary habits [21]. The linkages between the mentioned distinctions are complex, particularly because the relationships between some operate on the basis of reverse causality. The population living in the countryside or in small towns, for many reasons, has a lower level of education, lower income and more often remains unemployed. Unfavorable influence of the rural or small-town environment on the dietary patterns is confirmed by research [22]. Body weight was also examined as a factor which could reflect manner of food choices.

## 2. Materials and Methods

A questionnaire survey was carried out over the 2016–2017 period among parents of children born between 2003 and 2010, attending classes of year 1–6 in primary schools in Central Poland. This region was selected for the study because: It belongs to the province with the highest fertility rate and the highest birth rate [23]; it is a region where the health is subjectively assessed very positively, both by parents and adolescents [24]; and, it is characterized by having a gross domestic product (GDP) above the national average, which is often associated with a large variation in income of families, especially in different sub-regions [25].

Three sub-regions were selected from Central Poland—Kalisz, Konin and Pila—regions characterized by a similar number of pupils per 10,000 residents.

All data were collected using a semi-structured questionnaire containing 12 questions, including 4 open ones concerning knowledge of sources, functions and supplementation of vitamin D. Parents who correctly described at least one function of vitamin D were counted as parents who had knowledge about the importance of vitamin D on the human body. Participants who indicated at least one source of vitamin D were counted as parents who had knowledge about vitamin D sources. No answers, or incorrect answers, categorized them as parents without knowledge about vitamin D functions and sources. Other questions concerned place of residence, level of income, and level of education, as well as body weight and height of both children and parents. Parents had the opportunity to fill in the questionnaire independently and anonymously during the parents’ meetings organized at the schools their children attend.

The study was approved by the Scientific Research Ethics Committee with Participation of People at the Faculty of Human Nutrition Sciences and SGGW Consumption (No. 12/2017).

For statistical analyses, Statistica 13.1 software (StatSoft Inc., Tulsa, OK, USA) was used. The chi-squared test was performed to assess the existence of differences or dependencies between the analyzed qualitative data. Statistical significance of differences was estimated at *p* = 0.05.

## 3. Results

### 3.1. Total Population

The questionnaire was filled in by 783 parents, with a slight majority of city residents. All questionnaires were completed by mothers of children. Most often, mothers had a secondary education level, normal body mass (18.5–24.9 kg/m^2^, according to calculated BMI value), and monthly income per family in the range of PLN 2000–4000 net (Table 1). Mothers with higher education levels tended to more frequently live in the cities (51.5% vs. 26.4%). In the city, mothers more often had income above PLN 4000 net (39.0% vs. 19.1%). A better education level was also connected with better income and adequate body mass. Not all mothers answered questions concerning income, body mass and education level.

The number of respondents who were able to indicate at least one function and a source of vitamin D are listed in Table 2. The same table contains data concerning the frequency of supplementation in the studied group.

Each mother could indicate more than one vitamin D function and source. Most often, they declared that vitamin D contributes to the proper structure of bones and teeth (66%), supports immunity (19%), and positively influences the nervous system (8%). The respondents less frequently referred to its positive effects on the cardiovascular system (3%), proper growth of the body (3%), proper development (2%), muscle support (2%), improved condition of the skin (2%), or beneficial role in cancer prevention (2%). Most frequently mentioned sources were: Dairy products (40%), fish (37%), eggs (31%), and liver (4%). Mistaken responses were dominated by vegetable oils (5%), vegetables (5%) and fruits (3%).

### 3.2. Influence of Awarness about Vitamin D Functions and Sources on Frequence of Supplementation

Interestingly, not all mothers who were aware of vitamin D functions, and/or of limited dietary sources, supplemented it. However, as indicated in Table 3, awareness of both functionality and dietary sources had a significant influence on supplementation frequency.

### 3.3. Influence of Place of Residence

The relationship between knowledge about the function and sources of vitamin D, and its supplementation, combined with the place of residence of women, was also assessed (Table 4). Proper functions of vitamin D and its nutritional sources were known by more mothers living in the countryside. In addition, a higher frequency of vitamin D self-supplementation among women, and of vitamin D administration to children, was observed in the countryside. However, differences in the frequency of supplementation were not statistically significant.

### 3.4. Influence of Mothers’ Education Level

A significant relationship was observed between the level of mothers’ education and three of the four examined parameters (Table 5). Forty mothers did not respond to the question concerning level of education; therefore, the total population in this case was 740.

A majority of mothers with basic and vocational education were unable to indicate the proper functions of vitamin D, whereas in the group of mothers with secondary and higher education, this problem was much rarer. Knowledge about vitamin D functions and its nutritional sources rose with the level of mothers’ education. Better knowledge of functions and sources was associated with an increase in the frequency of supplementation in women and their children. Mothers with higher levels of education more often took supplements and gave them to their children. This effect was especially visible between the group of mothers with secondary education and that of mothers with primary and vocational education. However, this relationship is statistically significant for self-supplementation only. The effect of education level on child supplementation did not reach statistical significance.

### 3.5. Influence of Mothers’ Body Mass

The study also assessed the impact of mothers’ BMI on their knowledge of functions and sources, and their attitude to supplementation. Sixty-three mothers did not respond to the question concerning body mass and height; therefore, the total population in this case is 720. In spite of the fact that underweight mothers and those with a normal body mass were relatively more familiar with the sources and functions of vitamin D than overweight or obese mothers, these relationships were not found to be statistically significant. Moreover, the association between mothers’ body mass and the frequency of supplementation of parents and children did not achieve statistical significance (Table 6).

### 3.6. Influence of Monthly Net Income Per Family

A statistically significant relationship was observed only between the level of monthly income and the knowledge of the functions of vitamin D. This can be connected with the fact that mothers with higher incomes more often had better education levels. A trend of dependence between the higher net income of the family and better knowledge about nutritional sources of vitamin D was also observed. Contrary to this, income did not impact the self- and child supplementations. Ninety-nine mothers did not respond to the question concerning body mass and height; therefore, total population in this case was equal to 684 (Table 7).

## 4. Discussion

The importance of education in vitamin D deficiency prevention was supported in our study by the fact that mothers who were aware of vitamin D roles and dietary sources frequently used supplementation at a statistically significant higher level. Moreover, mothers’ education level was the only factor significantly influencing the level of knowledge about vitamin D food sources and functions, and the prevalence of its supplementation. Mothers’ higher education favored both state of knowledge about vitamin D and frequency of its self-supplementation. However, the effect of maternal education on child supplementation was weaker and nonsignificant. Thus, the frequency of supplementation was not affected by net income, mothers’ BMI, or the place of residence. Countryside residence supported the knowledge about vitamin D sources and functions, while income over PLN 4000 only supported awareness about functions.

The respondents were best at identifying the functions of vitamin D; 75% of them were able to name at least one of the functions of this vitamin and were aware of its importance for human health. High awareness about the effects of vitamin D on health of the skeleton was probably related to the wide coverage concerning this topic in Polish commercial media channels. Unfortunately, food sources were correctly indicated by less than half of mothers. Despite the fact that diet is not able to provide an adequate dose of vitamin D, it should be structured in such a way as to contribute to the reduction of deficiencies, especially when the time spent actively outdoors by children is limited. Unfortunately it is not because Polish children have low consumption of fish and seafood and in addition parents have insufficient knowledge about the basic dietary sources of vitamin D. In spite of a widespread information campaign on the problem of vitamin D deficiency in the population, the frequency of its supplementation is still very low. In the examined population, vitamin D was not supplemented among over 60% of children and over 70% of their mothers.

The state of knowledge concerning vitamin D among different age groups has been a subject of interest of many researchers on different continents. All cited authors stated that awareness of vitamin D sources and its role in the human body is insufficient. Those conclusions are in line with the results of our study.

Telephone interviews conducted among 547 middle-aged Chinese women living in Hong Kong showed that although 72.6% of respondents had heard about vitamin D, only 32.2% could indicate the role of this nutrient. Sources were correctly named by 38.4% of respondents. All results were lower than those obtained in our study. Just as for Polish parents, Chinese women mistakenly indicated vegetables as a source of vitamin D [26].

In Brisbane, Australia, an online survey was conducted among office workers. From 2867 participants who completed the questionnaire, nearly one-third indicated that they had no knowledge of the benefits of vitamin D, and this answer was more common in men than in women. Results in this case also were worse than those in our sample. In this research, approximately one-third of respondents who said they knew the sources of vitamin D indicated an incorrect food source [27]. Most participants of another Australian study felt that they knew less about the role of vitamin D in comparison to other vitamins. This was mainly attributed to comparatively limited media attention given to vitamin D compared to other vitamins [28]. Findings from other Australian surveys suggested that there was a limited awareness and understanding about vitamin D in the general population. This was much more evident than among Polish mothers, because 80% of participants in a study group were unable to name a health benefit of adequate vitamin D levels [29].

Knowledge, attitude and practice regarding vitamin D deficiency among female students was also assessed in Saudi Arabia. In this case, participants also had limited knowledge about vitamin D. In addition, limited sun exposure due to intense heat, cultural reasons for covering the body, and infrastructure, made sun synthesis difficult [30]. Polish pupils have also limited sun exposure, but it is caused by the climate not the culture. Other research in this region emphasized the necessity of education concerning vitamin D, similar to the findings of our study. One piece of research investigated whether knowledge, attitudes and behaviors related to vitamin D contributed to the prevalence of vitamin D deficiency among adults with and without coronary heart disease (CHD). In CHD sufferers, severe vitamin D deficiency was more prevalent, and total knowledge and supplementation of vitamin D less prevalent, compared to the control group. Seventy percent of the control group knew of vitamin D and half were aware of its role for healthy bones. Similar to Polish mothers, participants thought quite often that fruit and vegetables were vitamin D sources [31]. As in our study, another piece of research from Saudi Arabia demonstrated scarcity of knowledge in relation to dietary sources of vitamin D [32].

According to authors from Kuwait, the majority of study participants had limited knowledge, poor practices, and negative attitudes toward vitamin D problems, and therefore education intervention should be obligatory, which is similar to our conclusion [33]. Low knowledge of health effects and sources of vitamin D has also been observed in Canadian student studies. In a group of 1088 respondents, 37% were able to identify the role of vitamin D, and 26% were able to identify its sources [34]. An alarming result was obtained in a group of New Zealand athletes. Only 17% of 110 participants were able to identify sources of this vitamin other than the sun [35].

Insufficient knowledge about vitamin D and its sources was also reported by 71% of 116 pregnant women from distinct ethnic backgrounds (Caucasian Irish, Asian, Sub-Saharan African, or of Middle Eastern and North African background). Twenty-three percent of examined women did not know any source of vitamin D. Among those that were able to point out a source of vitamin D, 43% reported dairy foods and 23% mentioned, like Polish mothers, miscellaneous foods such as fruit and vegetables, soya products, and rice. The proportions of women who identified good sources of vitamin D were not significantly different across regions [36].

All cited articles emphasize the necessity of wider education concerning vitamin D sources and its functions for the human body. These findings are also applicable to Polish parents, who should widen their knowledge in this area. High awareness of vitamin D is essential in combating worldwide deficiencies.

## 5. Conclusions

A large-scale information campaign directed at parents about vitamin D’s role in the human body and its dietary sources should be conducted in Poland. A good solution could be to provide this type of information in social media or schools as a place of contact with parents. Short lectures could also be given or leaflets distributed; this should not only be during teacher-parent meetings. Raising awareness about vitamin D in Poland should be a key element in counteracting the widespread deficiency of this vitamin and its comorbidities in our country.

## Figures and Tables

**Table 1:** Characteristics of respondents’ sample (*n* = 783).

Environmental Factor		Number of Respondents	% of Respondents in Each Group
I. Place of residence	City	421	53.8%
	Village	362	46.2%
II. Mothers’ education level	Basic and vocational education	47	6.0%
	Secondary education	396	50.6%
	Higher education	297	37.9%
	Not declared	43	5.5%
III. Mothers’ body mass (according to BMI)	Underweight	32	4.1%
	Normal body mass	480	61.3%
	Overweight	165	21.1%
	obese	43	5.5%
	not declared	63	8.0%
IV. Net income per family	<2 K PLN	179	22.9%
	2–4 K PLN	301	38.4%
	4–6 K PLN	152	19.4%
	6–8 K PLN	32	4.1%
	>8 K PLN	20	2.6%
	Not declared	99	12.6%

**Table 2:** Knowledge about vitamin D function, nutrition sources and its supplementation popularity among respondents (*n* = 783).

Reviewed Factor	Total Number of Respondents	Number of Respondents Who Has Knowledge of/Use Supplements	% of Respondents
Knowledge about functions	783	580	74.1%
Knowledge about nutrition sources		389	49.7%
Self-supplementation		205	26.2%
Child supplementation		299	38.2%

**Table 3:** Popularity of self- and child supplementation among parents who are and are not aware of vitamin D dietary sources and functions (*n* = 783) (*p* = 0.05).

**Knowledge about Vitamin D Function**	**Total**	**Self-Supplementation**			**Child Supplementation**		
	**No.**	**No.**	**%**	**χ** **^2^** ***p***	**No.**	**%**	**χ** **^2^** ***p***
Yes	580	167	28.8%	7.9 0.004	246	42.4%	16.9 0.000
No	203	38	18.7%		53	26.1%	
**Knowledge about Vitamin D Sources**	**Total**	**Self-Supplementation**			**Child Supplementation**		
	**No.**	**No.**	**%**	**χ** **^2^** ***p***	**No.**	**%**	**χ** **^2^** ***p***
Yes	389	118	30.3%	6.9 0.008	169	43.4%	9.1 0.003
No	394	87	22.1%		130	33.0%	

**Table 4:** The influence of place of residence on knowledge and supplementation of vitamin D (*n* = 783) (*p* = 0.05).

Place of Residence	Total	Knowledge about Functions			Knowledge about Nutrition Sources			Self-Supplementation			Child Supplementation		
	No.	No.	%	χ^2^ *p*	No.	%	χ^2^ *p*	No.	%	χ^2^ *p*	No.	%	χ^2^ *p*
City	421	298	70.8%	5.1 0.023	192	45.6%	6.1 0.014	100	23.8%	2.8 0.096	156	37.1%	0.5 0.482
Village	362	282	77.9%		197	54.4%		105	29.0%		143	39.5%	

**Table 5:** The influence of level of mothers’ education on knowledge and supplementation of vitamin D (*n* = 740) (*p* = 0.05).

Mothers’ Education Level	Total	Knowledge about Functions			Knowledge about Nutrition Sources			Self-Supplementation			Child Supplementation		
	No.	No.	%	χ^2^ *p*	No.	%	χ^2^ *p*	No.	%	χ^2^ *p*	No.	%	χ^2^ *p*
Primary/vocational	47	20	42.6%	35.0 0.000	12	25.5%	16.7 0.000	3	6.4%	11.0 0.004	11	23.4%	5.1 0.078
Secondary	396	291	73.5%		192	48.5%		107	27.0%		158	39.9%	
Higher	297	244	82.2%		168	56.6%		87	29.3%		119	40.1%	

**Table 6:** The influence of mother’s body mass on knowledge and supplementation of vitamin D (*n* = 720) (*p* = 0.05).

Mother’s Body Mass (According to BMI Value)	Total	Knowledge about Functions			Knowledge about Nutrition Sources			Self-Supplementation			Child Supplementation		
	No.	No.	%	χ^2^ *p*	No.	%	χ^2^ *p*	No.	%	χ^2^ *p*	No.	%	χ^2^ *p*
Underweight (BMI < 18.5 kg/m^2^)	32	26	81.3%	5.0 0.169	17	53.1%	2.3 0.513	6	18.8%	1.8 0.618	11	34.4%	0.8 0.855
Normal (18.5–24.9 kg/m^2^)	480	369	76.9%		246	51.3%		136	28.3%		192	40.0%	
Overweight (25–29.9 kg/m^2^)	165	122	73.9%		82	49.7%		45	27.3%		65	39.4%	
Obesity (>30 kg/m^2^)	43	27	62.8%		17	39.5%		10	23.3%		15	34.9%	

**Table 7:** The influence of net income per family on knowledge and supplementation of vitamin D (*n*= 684) (*p* = 0.05).

Net Monthly Income Per Family	Total	Knowledge about Functions			Knowledge about Nutrition Sources			Self-Supplementation			Child Supplementation		
	No.	No.	%	χ^2^ *p*	No.	%	χ^2^ *p*	No.	%	χ^2^ *p*	No.	%	χ^2^ *p*
<2 K PLN	179	131	73.2%	12.7 0.012	76	42.5%	8.6 0.073	43	24.0%	2.8 0.598	69	38.5%	7.5 0.111
2–4 K PLN	301	213	70.8%		151	50.2%		75	24.9%		102	33.9%	
4–6 K PLN	152	130	85.5%		87	57.2%		42	27.6%		66	43.4%	
6–8 K PLN	32	25	78.1%		19	59.4%		11	34.4%		15	46.9%	
>8 K PLN	20	16	80.0%		11	55.0%		7	35.0%		11	55.0%	

## References

[1] Grineva E.N., Karonova T., Micheeva E., Belyaeva O., Nikitina I.L. (2013). Vitamin D deficiency is a risk factor for obesity and diabetes type 2 in women at late reproductive age. Aging.

[2] Marcinowska-Suchowierska E., Walicka M., Tałałaj M., Horst-Sikorska W., Ignaszak-Szczepaniak M., Sewerynek E. (2010). Vitamin D supplementation in adults—Guidelines. Endokrynol. Pol..

[3] Czech-Kowalska J. (2014). Current recommendations on vitamin D supplementation according to overall health consequences of vitamin D deficiency in pediatric population. Klin. Pediatr. Algorithms Ped..

[4] Hossein-Nezhad A., Holick M.F. (2013). Vitamin D for health: A global perspective. Mayo Clin. Proc..

[5] Christakos S., Dhawan P., Verstuyf A., Verlinden L., Carmeliet G. (2016). Vitamin D: Metabolism, molecular mechanism of action, and pleiotropic effects. Physiol. Rev..

[6] Czerwiński E., Borowy P., Kumorek A. (2012). Vitamin D and musculoskeletal system. Stand. Med. Pediatr..

[7] Kubis A.M., Piwowar A. (2015). The new insight on the regulatory role of the vitamin D3 in metabolic pathways characteristic for cancerogenesis and neurodegenerative diseases. Ageing Res. Rev..

[8] Burt M.G., Mangelsdorf B.L., Stranks S.N., Mangoni A.A. (2016). Relationship between Vitamin D status and autonomic nervous system activity. Nutrients.

[9] Gruber B.M. (2015). The phenomenon of vitamin D. Postepy Hig. Med. Dośw..

[10] Lerchbaum E., Obermayer-Pietsch B. (2012). Vitamin D and fertility: A systematic review. Eur. J. Endocrinol..

[11] Mohr S.B., Garland C.F., Gorham E.D., Garland F.C. (2008). The association between ultraviolet B irradiance, vitamin D status and incidence rates of type 1 diabetes in 51 regions worldwide. Diabetologia.

[12] Hyppönen E., Läärä E., Reunanen A., Järvelin M.R., Virtanen S.M. (2001). Intake of vitamin D and risk of type 1 diabetes: A birth-cohort study. Lancet.

[13] Garland C.F., Gorham E.D., Mohr S.B., Garland F.C. (2009). Vitamin D for cancer prevention: Global perspective. Ann. Epidemiol..

[14] McDonnell S.L., Baggerly C., French C.B., Baggerly L.L., Garland C.F., Gorham E.D., Lappe J.M., Heaney R.P. (2016). Serum 25-hydroxyvitamin D concentrations ≥40 ng/mL are associated with >65% lower cancer risk: Pooled analysis of randomized trial and prospective cohort study. PLoS ONE.

[15] Płudowski P., Konstantynowicz J., Jaworski M., Abramowicz P., Ducki C. (2014). Assessment of vitamin D status in Polish adult population. Stand. Med. Pediatr..

[16] Buczkowski K., Chlabicz S., Dytfeld J., Horst-Sikorska W., Jaroszyński A., Kardas P., Marcinkowska M., Siebert J., Tałataj M. (2013). Recommendations for vitamin D supplementation. Forum Med. Rodz..

[17] Institute of Medicine (2011). Dietary Reference Intakes for Calcium and Vitamin D.

[18] Płudowski P., Karczmarewicz E., Chlebna-Sokół D., Czech-Kowalska J., Dębski R., Dobrzańska A., Franek E., Głuszko P., Konstantynowicz J., Książyk J.B. (2013). Vitamin D supplementation in healthy population and risk groups of vitamin D deficiency—Practice guidelines for Central Europe 2013. Stand. Med. Pediatr..

[19] Sochocka L., Gruszka J. (2015). Methods chosen by parents of preschoolers to prevent vitamin D deficiency. Med. Środowiskowa.

[20] Wroński K., Bocian R. (2011). The patient’s knowledge about the role of vitamin D in chemoprevention of colorectal cancer. Nowa Med..

[21] Mazur J., Woynarowska B. (2004). Indicators of social inequalities for school-age children health surveys. Prz. Epidemiol..

[22] Babicz-Zielińska E., Schlegel-Zawadzka M., Wądołowska L., Przysławski J., Czarnocińska J. (2004). Influence of living place on the food preferences and eating frequency. Bromatol. Chem. Toksykol..

[23] Statistic Poland (2017). Yearbook of Poland.

[24] Statistic Poland (2015). Health and Health Care in 2015.

[25] Statistic Poland (2015). Gross Domestic Product—Regional Accounts in 2015.

[26] Kung A., Lee K. (2006). Knowledge of vitamin D and perceptions and attitudes toward sunlight among Chinese middle-aged and elderly women: A population survey in Hong Kong. BMC Public Health.

[27] Vu L., van der Pols J., Whiteman D., Kimlin M., Neale R. (2010). Knowledge and Attitudes about Vitamin D and Impact on Sun Protection Practices among Urban Office Workers in Brisbane, Australia. Cancer Epidemiol. Biomark..

[28] Bonevski B., Bryant J., Lambert S., Brozek I., Rock V. (2013). The ABC of Vitamin D: A Qualitative Study of the Knowledge and Attitudes Regarding Vitamin D Deficiency amongst Selected Population Groups. Nutrients.

[29] Janda M., Youl P., Bolz K., Niland C., Kimlin M. (2010). Knowledge about the health benefits of vitamin D in Queensland Australia. Prev. Med..

[30] Christie F., Mason L. (2011). Knowledge, attitude and practice regarding vitamin D deficiency among female students in Saudi Arabia: A qualitative exploration. Int. J. Rheum. Dis..

[31] Aljefree N., Patricia L., Faruk A. (2017). Knowledge and attitudes about vitamin D, and behaviors related to vitamin D in adults with and without coronary heart disease in Saudi Arabia. BMC Public Health.

[32] Aljefree N., Lee P., Faruk A. (2017). Exploring Knowledge and Attitudes about Vitamin D among Adults in Saudi Arabia: A Qualitative Study. Healthcare.

[33] Al Bathi B., Al Zayeda K., Al Qenai M., Makboul G., El-Shazly M. (2012). Knowledge, attitude and practice of patients attending primary care centers toward vitamin D in Kuwait. Alex. J. Med..

[34] Boland S., Irwin J.D., Johnson A.M. (2015). A Survey of University Students’ Vitamin D–Related Knowledge. J. Nutr. Educ. Behav..

[35] Walker N., D Love T., Baker D., Healey P., Haszard J., Edwards A., Black K. (2014). Knowledge and attitudes to vitamin D and sun exposure in elite New Zealand athletes: A cross-sectional study. J. Int. Soc. Sports Nutr..

[36] Toher C., Lindsay K., McKenna M., Kilbane M., Curran S., Harrington L., Uduma O., McAuliffe M. (2014). Relationship between vitamin D knowledge and 25-hydroxyvitamin D levels amongst pregnant women. J. Hum. Nutr. Diet..

